# THE EFFECT OF AGE ON EMOTION REGULATION PATTERNS IN DAILY LIFE: FINDINGS FROM AN EXPERIENCE SAMPLING STUDY

**DOI:** 10.1093/geroni/igad104.0983

**Published:** 2023-12-21

**Authors:** Mai Mikkelsen, Mia O’Toole, Emma Elkjær, Mimi Mehlsen

**Affiliations:** Aarhus University, Aarhus C, Midtjylland, Denmark; Aarhus University, Aarhus C, Midtjylland, Denmark; Aarhus University, Aarhus C, Midtjylland, Denmark; Aarhus University, Aarhus C, Midtjylland, Denmark

## Abstract

The aim of the present experience sampling study was to examine how age affects emotion regulation patterns and their impact on well-being in daily life. A sample of 406 adults (age range: 18-81, 62.8 % female) were prompted five times a day for seven days to rate their momentary emotions, emotion regulation strategy use, and emotion regulation strategy effectiveness. Based on these ratings, indicators of emotion regulation variability and differentiation were calculated. Well-being outcomes included daily positive and negative emotions as well as symptoms of anxiety and depression assessed at baseline. The findings revealed reduced emotion regulation variability with age and a negative association between emotion regulation variability and well-being. There was no effect of age on emotion regulation effectiveness or differentiation. Emotion regulation effectiveness was associated with more positive and less negative daily emotions, and this association was stronger for young adults than older adults. The findings may indicate that older adults rely on a smaller set of effective emotion regulation strategies that are adapted to their specific context and resources, leading to reduced variability in emotion regulation and ultimately greater well-being. Concerning emotion regulation effectiveness, the findings suggest that effectiveness is less important for well-being in daily life for older adults, possibly because well-being is determined by other factors (e.g., less frequent and more predictable stressors) in older age.

